# Impact of Sample Pretreatment and Extraction Methods on the Bioactive Compounds of Sugar Beet (*Beta vulgaris* L.) Leaves

**DOI:** 10.3390/molecules27228110

**Published:** 2022-11-21

**Authors:** Peyman Ebrahimi, Dasha Mihaylova, Christine Mayr Marangon, Luca Grigoletto, Anna Lante

**Affiliations:** 1 Department of Agronomy, Food, Natural Resources, Animals, and Environment—DAFNAE, Agripolis, University of Padova, 35020 Legnaro, Italy; 2 Department of Biotechnology, University of Food Technologies, 26 Maritza Blvd., 4002 Plovdiv, Bulgaria

**Keywords:** food by-products, ultrasound-assisted extraction (UAE), lyophilization, green technologies, polyphenol oxidase, enzymatic browning, polyphenols, freeze-drying, amino acids, fatty acids

## Abstract

To find the most optimal green valorization process of food by-products, sugar beet (*Beta vulgaris* L.) leaves (SBLs) were freeze-dried and ground with/without liquid nitrogen (LN), as a simple sample pretreatment method, before ultrasound-assisted extraction (UAE) of polyphenols. First, the water activity, proximate composition, amino acid (AA) and fatty acid (FA) profiles, and polyphenol oxidase (PPO) activity of dried and fresh SBLs were evaluated. Then, conventional extraction (CE) and UAE of polyphenols from SBLs using water/EtOH:water 14:6 (*v*/*v*) as extracting solvents were performed to determine the individual and combined effects of the sample preparation method and UAE. In all the freeze-dried samples, the specific activity of PPO decreased significantly (*p* ≤ 0.05). Freeze-drying significantly increased (*p* ≤ 0.05) the fiber and essential FA contents of SBLs. The FA profile of SBLs revealed that they are rich sources of oleic, linoleic, and α-linolenic acids. Although freeze-drying changed the contents of most AAs insignificantly, lysine increased significantly from 7.06 ± 0.46% to 8.32 ± 0.38%. The aqueous UAE of the freeze-dried samples without LN pretreatment yielded the most optimal total phenolic content (TPC) (69.44 ± 0.15 mg gallic acid equivalent/g dry matter (mg GAE/g DM)) and excellent antioxidant activities. Thus, combining freeze-drying with the aqueous UAE method could be proposed as a sustainable strategy for extracting bioactive compounds from food by-products.

## 1. Introduction

As the population of the world is growing sharply, providing food security to meet the optimal dietary needs of consumers has become a vital issue over the past few decades. Thus, it is necessary to find new sustainable food supplies [1,2]. On the other hand, the increased food production arising from this overpopulation leads to a noticeable generation of by-products, which may cause many environmental concerns [3]. However, these by-products could be considered nutritional resources as they possess high quantities of fiber, minerals, antioxidants, proteins, etc. [4]. Therefore, not only does the valorization of these by-products provide health benefits to consumers, but it can also increase global food security [5,6].

Sugar beet (*Beta vulgaris* L.) is one of the most prominent edible plants cultivated in Europe, where approximately 200 million tons of this plant are harvested annually. The leaves of this crop remain mostly unexploited as a by-product, of which 120 million tons per year are produced only in Europe [7]. Sugar beet leaves (SBLs) are more nutritious than beetroot, as they are rich in essential amino acids (AAs) and fatty acids (FAs), proteins, and polyphenols. Therefore, they could be considered a potent source of bioactive compounds, making them a potential food matrix to further analyze their valorization capacity [8,9,10]. Thus, the efficient and sustainable disposal of SBLs needs to be evaluated following the full-component utilization concept proposed by Zhang et al. (2021) [11]. However, it has been reported that there is high polyphenol oxidase (PPO) activity in SBLs [12], which may decrease the efficiency of extracting polyphenols [6]. In addition, the high moisture content of SBLs makes them perishable and difficult to store. Thus, it is required to apply a pretreatment on them before the extraction of bioactive compounds [13]. Drying the plant tissues is often suggested before the extraction process to reduce the moisture and overall deterioration of bioactive compounds [14].

**Figure 1:**
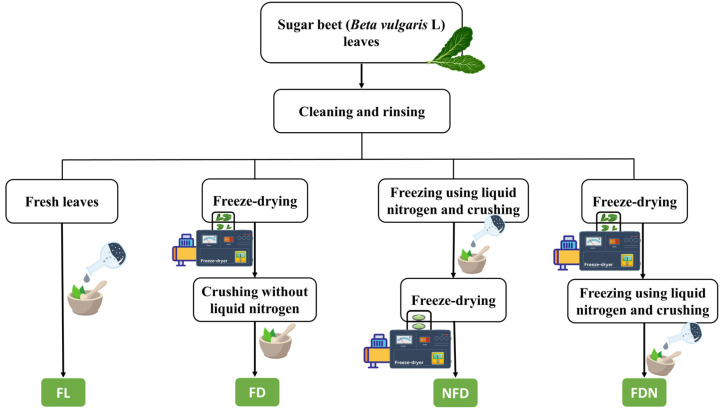
Preparation of samples with four different simple methods. FL: Fresh sugar beet leaves (SBLs); FD: Lyophilized SBLs ground without liquid nitrogen (LN); NFD: Lyophilized SBLs ground with LN before freeze-drying; FDN: Lyophilized SBLs ground with LN after freeze-drying.

The significance of pretreating samples has mostly been neglected in experimental designs. Finding faster, cheaper, and more environmentally friendly pretreatments is a determining factor in the valorization processes [15]. Freeze-drying or lyophilization is one of the mild procedures for removing water, which is proven to be the most optimal technique for preserving the sensitive compounds of food because of lower temperature and pressure, and the absence of water [16]. Therefore, it could be an appropriate treatment to overcome the problems stemming from the high moisture content of SBLs. The low temperature in this process maintains nutrients and bioactive compounds optimally and controls the undesirable effects of the Maillard reaction [16] and enzymatic browning [17]. However, the freeze-drying process could be very energy-consuming [18], and in some cases, it deteriorates the content of polyphenols [19,20]. Therefore, it is necessary to find a solution to control these problems. A possible option for overcoming the problems of freeze-drying is grinding the samples under liquid nitrogen (LN) before the drying process. In this method, the food matrix, which is ground into small particles, is freeze-dried [21]. LN is used for many cooling and cryogenic applications and boils at −195.80 °C in atmospheric pressure. Grinding plant tissues with LN using mortar and pestle is a widely utilized approach for recovering intracellular compounds [22]. It is crucial to appraise the impact of LN before and after freeze-drying on the bioactive compounds present in food by-products as a pretreatment before the extraction. Thus, the sample treatment method was designed as shown in Figure 1.

In addition to the sample preparation, selecting a proper extraction method is another key factor for achieving high yields of bioactive compounds, together with reducing the amount of solvent, and saving time and energy. Since conventional extraction (CE) methods, such as maceration, have many drawbacks, an attractive alternative is the ultrasound-assisted extraction (UAE) method, which is considered a green technology [13]. In the UAE method, the pressure and cavitation of ultrasonic waves lead to the disruption of the plant cell membrane, which results in the release of target components [23]. To our knowledge, there is no research investigating the combined effect of freeze-drying with LN treatment and UAE on the quantity and quality of bioactive compounds.

Therefore, this study aimed to evaluate the individual and combined effects of the sample preparation method and UAE on the bioactive compounds of SBLs. It was anticipated that the proper procedure of sample preparation and extraction conditions could positively impact the efficiency of the results. Therefore, proximate composition, water activity, and AA and FA profiles were first analyzed to better elaborate the chemical properties of different treatments of SBLs. Secondly, the PPO activity of SBLs was investigated to understand its connection with total phenolic content (TPC) and the antioxidant activity of the phenolic extracts, which were evaluated at the final stage of this project.

## 2. Results and Discussion

### 2.1. Proximate Composition and Water Activity of SBLs

The concentration of simple components in the plant tissues could have a noticeable impact on the freeze-drying process. For instance, high lipid content may decrease the quality of lyophilized products [21]. Table 1 shows the proximate composition and water activity of SBLs. There is no significant difference (*p* > 0.05) among the ash contents of different treatments. Various quantities of ash have been reported for SBLs in the literature, such as 17.76 g/100 g dry matter (DM) [24], 16.40 g/100 g DM [25], and 20.80 g/100 g DM, which are higher than the results of the present paper [26]. This could be due to the different chemical compositions of sugar beets with various agronomic traits, harvest time, and environmental factors (e.g., the geographical region, climate, and soil specifications) [24].

Since water content highly impacts the degradation of sensitive compounds, knowing the content of moisture and water activity of raw material is essential [27]. The results show that there are significant differences (*p* ≤ 0.05) between the moisture content and water activity of fresh SBLs (FL) and other treatments. The lower moisture content in lyophilized SBLs ground with LN before freeze-drying (NFD) could be related to the smaller size of leaves’ particles as water is released more readily from ground leaves. Moreover, the freezing rate in LN pretreatment significantly influences ice formation and determines the drying rate. When LN is used to freeze samples, the liquid changes to ice rapidly with a higher freezing rate and smaller crystal size [28], and the number of ice crystals increases. The size of crystals can affect the rate of drying [21]. Sublimation of fast-frozen food with small-sized ice crystals occurs rapidly in the first drying stage of the freeze-drying [18]. Based on these, in LN-pretreated samples (i.e., NFD), the moisture content decreases more efficiently. Alfaro et al. (2018) reported the same decrease in moisture content when they evaluated the effect of LN pretreatment on the osmotic dehydration of blueberries [29]. Since the freeze-drying method is very energy-consuming and requires a long drying period [18], the decrease in the moisture content by pretreating samples with LN could decrease the time required for drying and consequently, reduce the energy needed for the freeze-drying process.

The decrease in water activity after freeze-drying could be considered a beneficial factor for preserving bioactive compounds because lower water activities result in an increased viscosity preventing the mobility of compounds and chemical reactions [30]. However, Maldonado-Astudillo et al. (2019) reported that polyphenols are more extractable in higher water activities, although the changes in the phenolic content are minor in low water activities [31].

According to the results, there was a significant decrease in the crude protein content of all the freeze-dried SBLs (*p* ≤ 0.05). Since freeze-drying causes stresses such as exposure to ice-water interfaces, cold denaturation, and freeze-concentration, it can reduce the content of protein in plant tissues [32]. Moreover, this decrease could result from an overestimation of the protein content of FL as the Kjeldahl method uses a nitrogen-to-protein conversion factor, considering most of the nitrogen in the leaves as protein. Therefore, different amounts of non-protein nitrogen in samples cause this overestimation [33]. After all, it should be noted that the quality of proteins is more important than their quantity. Thus, it is necessary to characterize the AA profile of SBLs to specify the protein quality after freeze-drying.

Despite the decrease in protein content after freeze-drying, the crude protein content in SBLs is still high compared to the literature. The protein content of the SBLs on the basis of DM has been reported as 24.02 g/100 g [24], 22.8 g/100 g [25], and 19.4 g/100 g [34]. All these quantities are lower than the protein content found in SBLs in the present paper. The high protein content of SBLs makes them a promising source of sustainable vegetal protein, meeting the requirements of vegetarian and vegan people. Furthermore, the consumption of plant proteins could reduce cardiovascular diseases [35]. For all adults above 18 years old, the recommended dietary allowance of proteins is 0.83 g of protein/kg body weight/day [36]. Therefore, according to the total protein measured in the present paper, consumption of 2.89 g DM of FDN/kg body weight/day can meet the recommended protein intake of the body.

There is a significant increase (*p* ≤ 0.05) in the fiber content of all the freeze-dried SBLs. Most parts of the fiber found in sugar beet are soluble pectin and insoluble hemicellulose, with small quantities of lignin and cellulose [37]. Therefore, it is possible that SBLs also have the same constituent. Fernandez et al. (2017) [38] reported that the fiber content in the leaves of *Beta vulgaris* L. is approximately 2.93 g/100 g, almost identical to the results obtained in the present paper. Dietary fibers contain carbohydrate polymers with some non-carbohydrate components showing many health benefits, including the inhibition of obesity, diabetes, stroke, hypertension, and coronary heart disease [39]. Therefore, the high fiber content in the SBLs could have health benefits.

Moreover, fiber and polyphenols in plant-based food are essential in preventing metabolic alterations. When fiber and polyphenols reach the colon, they are fermented by the microbiota, resulting in the generation of short-chain fatty acids. These fatty acids regulate food intake and improve the expression of anorectic hormones in the colon [40]. It is reported that the fiber concentrations of sugar beet are practical in enhancing the rheological properties of meat products, resulting in acceptable structural or textural characteristics [37]. Moreover, Asadi et al. reported that the enrichment of cookies with beetroot leaves powder increased their crude fiber content from 0.78 ± 0.33% to 12.82 ± 0.32 [41]. Therefore, SBLs could be used in formulating functional foods with high health benefits for consumers.

Abdo et al. (2020) reported that the crude lipid content of beetroot leaves was 0.43 ± 0.04 g/100 g DM [42]. Furthermore, Kiskini et al. (2016) reported that SBLs aged three months have a lipid content of 4.20 g/100 g [25]. Since the leaves were going to be subjected to the extraction of polyphenols in the next stage of the project, estimating the lipid content is essential because the first step in extracting polyphenols is the removal of lipids (defatting) from the food matrix [27]. In the present paper, the lipid content of raw material is low (1.16 ± 0.05 g/100 g), and there was no need to remove lipids from the leaves before the extraction of polyphenols. Even though freeze-drying decreased the fat content of SBLs significantly (*p* ≤ 0.05), they had significantly higher lipid content (*p* ≤ 0.05) when they were pretreated with LN. However, this small increase could be beneficial to consumers as it has been reported that lipids can interact with polyphenols and protect them in their passage through the gastrointestinal tract, making it easy to deliver polyphenols to the lower parts of the gastrointestinal tract [43]. Thus, the lipid content in the SBLs is beneficial in the absorbance of polyphenols into the body.

### 2.2. Amino Acid Profile of Fresh and Lyophilized Leaves

AAs are categorized as nonessential and essential based on the ability of the body to synthesize them. The human body cannot synthesize essential AAs, and the only source for these AAs is dietary protein. Moreover, some nonessential AAs (e.g., arginine, cysteine, glutamine, glycine, proline, and tyrosine) could become essential in conditions such as premature birth. Thus, to overcome the deficiency of AAs and for the proper functioning of the body, it is crucial to consume dietary proteins containing all AAs [36].

**Figure 2:**
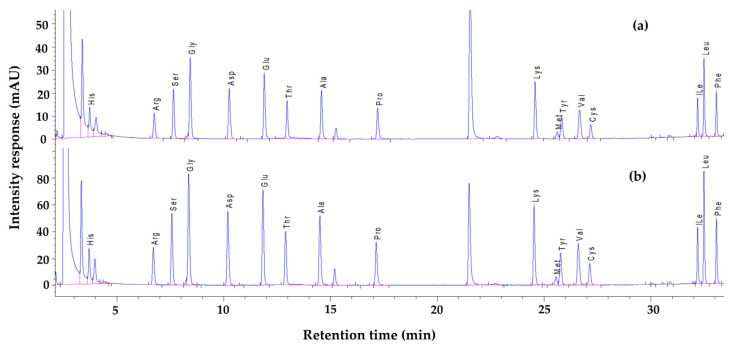
Representative chromatograms corresponding to the AA profiles of (**a**) fresh SBLs and (**b**) freeze-dried SBLs. Ala: alanine; Arg: arginine; Asp: aspartic acid; Glu: glutamic acid; Gly: glycine; His: histidine; ILe: isoleucine; Leu: leucine; Lys: lysine; Met: methionine; Phe: phenylalanine; Pro: proline; Ser: serine; Thr: threonine; Tyr: tyrosine; Val: valine; Cys: cysteine + cystine.

SBLs possess a balanced amino acid profile. The presence of essential AAs, including threonine, leucine, phenylalanine, isoleucine, valine, lysine, and methionine, reveals the particular nutritional quality of SBLs [24]. Table 2 shows the AA profile of SBLs treated with different methods. Figure 2 shows the representative chromatograms corresponding to the AA profiles of freeze-dried and fresh SBLs. The results show that SBLs contain a significant number of essential AAs, such as threonine, valine, phenylalanine, isoleucine, leucine, lysine, and methionine. Furthermore, glutamic acid has the highest concentration. This compound could be used as a food additive in the form of monosodium glutamate, a well-known flavor enhancer [24]. Therefore, the powder of SBLs could be used as a flavor enhancer as it has a high quantity of glutamic acid.

Most of the changes in the AA content of the leaves were insignificant (*p* > 0.05) after the freeze-drying and LN treatments. However, there were some significant increases in the content of some important AAs, including aspartic acid, lysine, valine, and isoleucine. The increase in the content of lysine after freeze-drying is a positive result as it is an essential AA, and its recommended intake ranges from 30 to 64 mg/kg body weight/day in different age groups [36]. Moreover, valine and isoleucine have positive effects on muscle development. Aspartic acid is also vital in synthesizing arginine, lysine, methionine, threonine, and isoleucine [24]. Since SBLs are rich sources of these AAs, they could be used as food supplements.

### 2.3. Fatty Acid Profile of SBLs

FAs are regarded as one of the most crucial nutrients for humans, and they are categorized according to the presence or absence of double bonds as saturated (SFA), monounsaturated (MUSFA), and polyunsaturated fatty acids (PUSFA) [44].

Table 3 summarizes the FA profile of fresh and treated SBLs. The results show some significant increases in short-chain and medium-chain SFAs in the freeze-dried and LN-treated samples. Although SFAs are correlated to some metabolic diseases, it is also widely acknowledged that their metabolic impacts differ according to their chain length. For instance, while long-chain SFAs are known to cause metabolic diseases, medium-chain SFAs have beneficial effects in promoting metabolism [45]. Many FAs, such as lauric, linoleic, and myristic acids are known to have antibacterial and antifungal properties [44]. Therefore, it could be claimed that the health benefits of freeze-dried SBLs are greater than those of fresh SBLs as the short-chain SFAs are generally used for their antibacterial, antiviral, and antioxidant properties, as human immunity enhancers and digestion improvers, and for other health-benefiting functions [46].

Freeze-drying increased the essential FAs (i.e., linoleic acid and α-linolenic acid) significantly (*p* ≤ 0.05). As anticipated, the results show that SBLs are a rich source of omega-3 and omega-6 which are the principal constituents of the membrane glycerolipids and the photosynthetic tissues of leaves [47]. Similar to the present paper, it was also reported that dried red beet leaves contain critical polyunsaturated FAs, such as linoleic acid and α-linolenic acid [10]. Similarly, Shukla et al. (2018) reported that *Cassia tora* leaves possess a high quantity of essential FAs [44]. Consuming foods rich in α-linolenic acid can prevent disorders such as prostate cancer, rheumatoid arthritis, colon cancer, coronary diseases, and brain cancer [10]. Moreover, co-supplementing omega-3 FAs and vitamin E can reduce the very low-density lipoprotein (VLDL) [48]. Thus, SBLs could have many anti-disease effects on consumers.

However, arachidonic acid decreased significantly (*p* ≤ 0.05) after the freeze-drying. The change in the unsaturated FAs results from the attachment of oxygen atoms, the disruption of fatty acid molecules at the site of double bonds, the breakdown of these unstable intermediates, and the formation of oxygen derivatives of organic compounds [49]. In addition, the results show that SBLs are rich in oleic acid content. MUSFAs such as oleic and palmitoleic fatty acids can reduce the level of low-density lipoproteins (LDL) and maintain the level of high-density lipoproteins (HDL) constant, which is vital to controlling blood levels of cholesterol in the plasma [50].

### 2.4. Polyphenol Oxidase Activity of SBLs

The enzymatic browning of plant foods results from mechanical and physical stresses happening in the processing and storage of food products. This reaction is mainly driven by PPO, an intracellular o-diphenol oxidase [51]. PPO is a copper-containing oxidoreductase enzyme that uses polyphenols as a substrate in the enzymatic browning reaction [5]. This reaction occurs when the cell structure ruptures, and the subsequent interaction between PPO and polyphenols causes a color change and a decrease in antioxidant activity, leading to the deterioration of the nutritional properties of food matrices. In addition, the reaction between PPO and polyphenols gives rise to their oxidation, resulting in a decrease in TPC, which negatively impacts the extraction process of polyphenols from food by-products. Therefore, finding a suitable method for reducing the disadvantages derived from PPO is crucial [6]. Partial or full cessation of PPO activity could be achieved by removing the enzyme substrates (e.g., polyphenols), controlling the pH of the medium, adding sodium sulfite and ascorbic acid, or thermal inactivation [52]. Some studies have reported that freeze-drying can deactivate the PPO enzyme [53].

**Figure 3:**
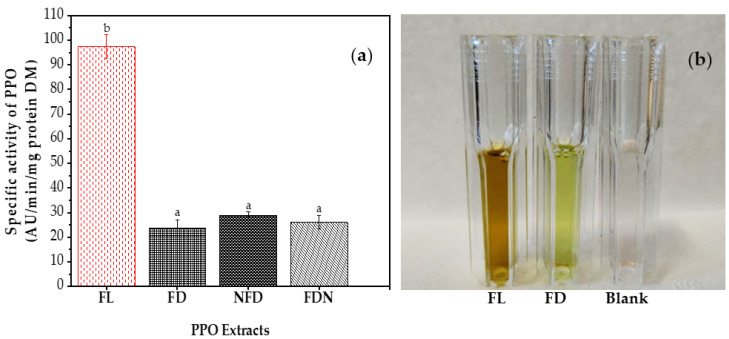
(**a**) Specific activity of polyphenol oxidase (PPO) in different SBLs. (**b**) Color change of PPO extracts after 180 s in the presence of catechol. Data are presented as mean ± SD (*n* = 3). Different letters show that there is a significant difference (*p* ≤ 0.05), according to one-way ANOVA and Tukey’s Honestly Significant Difference (HSD) tests.

Figure 3a indicates the specific activity of PPO. Freeze-drying significantly decreased (*p* ≤ 0.05) the specific activity of PPO in SBLs, causing a reduced enzymatic browning in the PPO extract of freeze-dried SBLs, as shown in Figure 3b. This inactivation is consistent with the results obtained by Valadez-Carmona et al. (2017) [53]. They reported that freeze-drying reduced the activity of PPO by 52% in cacao pod husks. The reduction in PPO activity could result from an increase in the H^+^ concentration during freezing, which can change the pH value by three units, reducing the stability of PPO [30]. The reduction in PPO content may positively affect the extraction of polyphenols from food by-products since the reaction between PPO and polyphenols gives rise to their oxidation resulting in a decrease in TPC [6]. Therefore, the decrease in PPO obtained by the freeze-drying method can improve the extraction efficiency of polyphenols. The changes among the freeze-dried SBLs were insignificant, although there was a minuscule increase in the PPO activity of samples treated with LN. PPO in plant cells exists in both free and bound states. The free PPO mostly exists in the cell serum, and the bound PPO is mainly present in chloroplasts and vacuoles. These two states of PPO can be changed by freezing, as it can partially inactivate the free state enzymes, while part of the bound state enzymes in the cell wall and other organelles can be released and converted into the free state due to freezing [28].

### 2.5. Total Phenolic Content (TPC) and Antioxidant Activity of Extracts

In many studies, plant leaves are considered a potential source of bioactive compounds providing health benefits to consumers [3,54,55,56]. Polyphenols are one of these biologically active substances possessing antibacterial, antifungal, anti-inflammatory, and anti-cancer properties [5]. SBLs are rich in polyphenols with high antioxidant activity [57]. De Castro et al. (2019) reported that freeze-dried red beet leaves are a potential dietary food product that can prevent obesity [58]. Lorizola et al. (2018) investigated the impact of phenolic compounds of SBLs on oxidative liver damage in mice fed with a high-fat diet, and they reported that dried SBLs decreased the adverse impacts of this diet on lipid metabolism, reduced fasting blood glucose levels, lowered cholesterol levels, and decreased glutathione peroxidase and glutathione reductase activities in mice [59]. Moreover, consuming SBLs could be beneficial due to their high polyphenol content because polyphenols can inhibit acetylcholinesterase, which is an enzyme linked to the development of Alzheimer’s disease. Indeed, acetylcholinesterase is involved in the metabolic hydrolysis of acetylcholine at cholinergic synapses in the central and peripheral nervous system and deteriorates the level of the neurotransmitter acetylcholine. Polyphenols block the action of acetylcholinesterase and thus enhance the brain’s level of acetylcholine [60].

**Figure 4:**
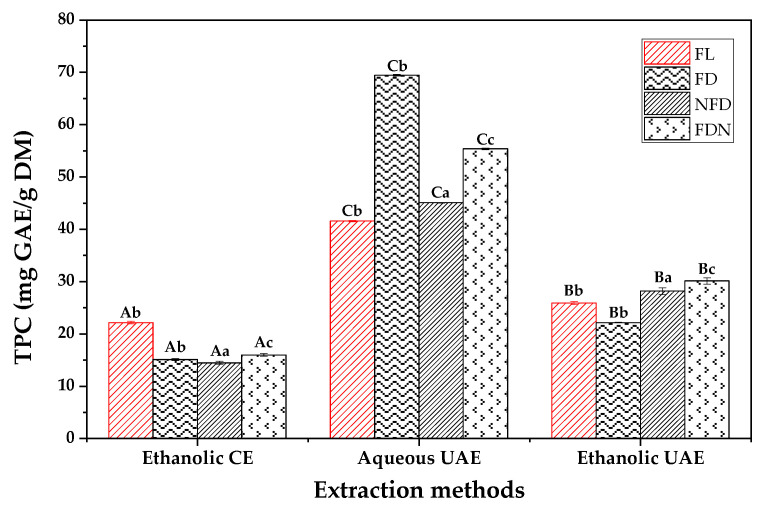
Total phenolic content (TPC) of phenolic extracts obtained from SBLs. Data are presented as mean ± SD (*n* = 3). Different capital letters indicate significant differences in the extraction method, and different small letters show significant differences in the sample preparation method (*p* ≤ 0.05), according to two-way ANOVA and Tukey’s HSD test.

Figure 4 illustrates the TPC of the phenolic extracts obtained from the SBLs using the CE and UAE (i.e., ethanolic and aqueous) methods. The results from the two-way ANOVA showed that the extraction method and sample preparation method both have significant effects (*p* ≤ 0.05) on the results. Although the aqueous UAE of lyophilized SBLs ground without LN (FD) had the highest TPC (69.44 ± 0.15 mg gallic acid equivalent/g dry matter (mg GAE/g DM)), it had the lowest TPC (15.12 ± 0.19 mg GAE/g DM) when extracted using ethanolic CE. These results show that the extraction conditions, including the sample preparation method and extracting solvent type, are important for achieving the optimal results. It is reported that the content of polyphenols in food by-products may be affected by the freeze-drying technique [61]. Tan et al. (2020) showed that freeze-drying is the most optimal drying method for maintaining total polyphenols and their antioxidant activities [62]. Moreover, Esteban-Lustres et al. (2022), comparing the effect of freeze-drying and conventional drying treatments before UAE, reported that the freeze-drying procedure improves the extraction yield of polyphenols from food waste [13]. On the other hand, some research has reported the negative effect of this process on polyphenols [19,20].

Sakakibara et al. (2003) pretreated fresh vegetables and fruits using nitrogen before freeze-drying, and they reported that these samples had a good phenolic recovery efficiency ranging from 68 to 92% [63]. The reason for the higher TPC in LN-pretreated samples could be related to the higher freezing rate of these samples compared to the other samples frozen in the refrigerator. Rapid freezing of food matrices using LN has the advantages of a high heat transfer coefficient, fast freezing rate, short freezing time, and the formation of small ice crystals, which can result in better preservation of nutrients [28,64]. However, this was true only when we used ethanol as the extracting solvent.

The TPCs of all freeze-dried samples in the present paper are noticeably higher than the results obtained by Duck et al. (2022) when they extracted polyphenols using UAE from SBLs. The highest TPC reported by them was 17.89 ± 0.38 mg GAE/g DM, which was extracted from dried SBLs with a 2:100 solid:liquid ratio (*w*/*v*), 100% amplitude, and 9 min extraction time [8]. Therefore, it could be confirmed that the method proposed in the present paper is a reliable procedure with good efficiency. Moreover, Abdo et al. (2020) reported that ethanolic extracts of beetroot leaves recovered instantly had a TPC of 8.54 mg GAE/g DM [42]. The lower yield of TPC in their work could be related to the extraction method they used to recover polyphenols.

Arjeh et al. (2022) extracted polyphenols from the flesh and peel of freeze-dried sugar beet root using the UAE method and methanol and ethanol as solvents, and the highest TPC yielded was 19.70 ± 1.10 mg GAE/g DM in the methanolic extract of its peel, which is much lower than the TPC obtained by the aqueous UAE of FD in the present paper [65]. Goyeneche et al. (2020) recovered polyphenols from beetroot leaves using supercritical CO_2_ extraction, and the best result obtained was a TPC of 3.37 mg GAE/g DM and DPPH of 1.45 mg Trolox equivalent/g dry matter (mg TE/g DM) [56]. Bengardino et al. (2019) optimized the recovery of polyphenols from beet leaves and they reported a TPC of 31.70 mg GAE/g DM [55]. Therefore, the SBLs have more phenolic content than their root. Ebrahimi et al. (2022) reported that the phenolic extracts of SBLs are rich in pyrogallol, 4-hydroxybenzoic acid, and syringic acid [57].

**Figure 5:**
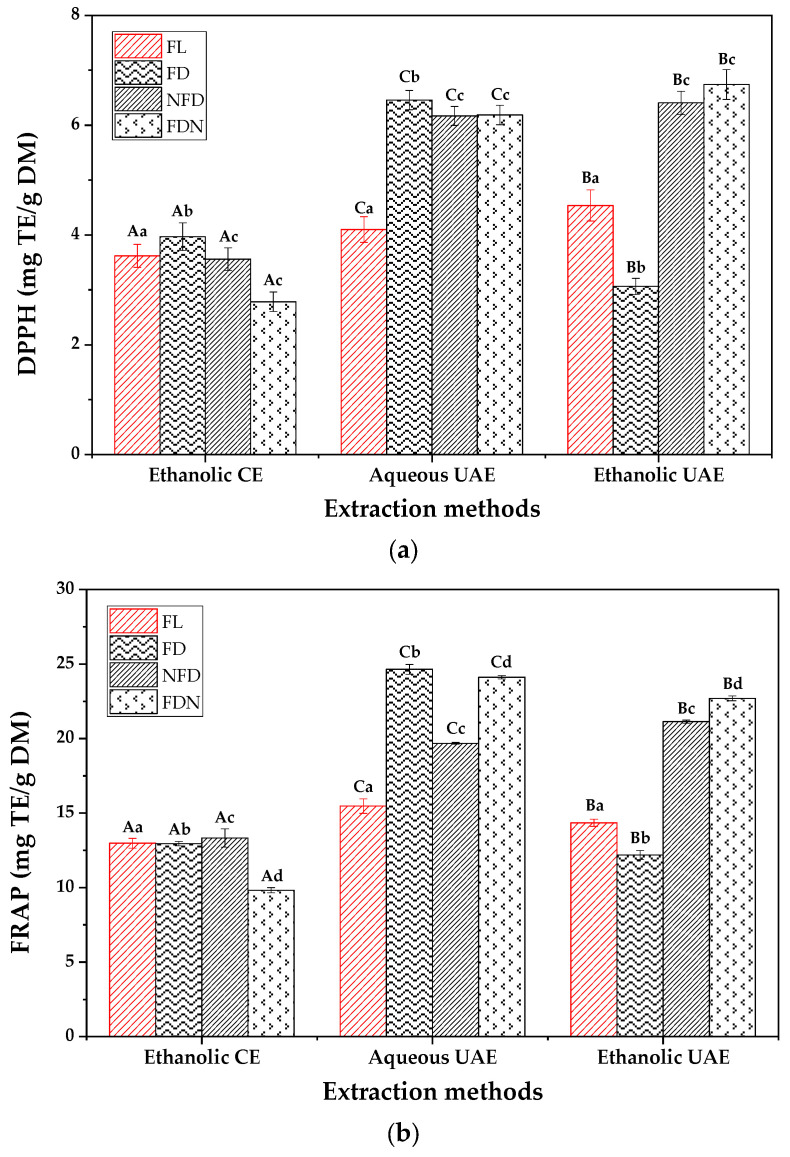
DPPH (**a**) and FRAP (**b**) of phenolic extracts obtained from SBLs. Data are presented as mean ± SD (*n* = 3). Different capital letters indicate significant difference in the extraction method, and different small letters indicate significant difference in the sample preparation method (*p* ≤ 0.05), according to two-way ANOVA and Tukey’s HSD tests.

Figure 5 shows the antioxidant activity of the phenolic extracts of the SBLs. The antioxidant activity is highly connected to the quantity of polyphenols. However, it should be noted that the type and quality of phenolic compounds are more responsible for the amount of antioxidant activity than their quantity [5]. According to the results, UAE increased the TPC and antioxidant activities in almost all the samples. The reason for this could be the ability of the UAE to increase the penetration of the solvent into the solid matrix and facilitate the mass transfer rate of phenolic compounds from the food matrix to the solvent [66]. Moreover, UAE could cause a decrease in the PPO content of food matrices by changing the environment of the enzyme, disrupting Vander Wall binding and hydrogen bonding in the enzyme, which results in loss of enzyme activity [67]. This can increase the extractability of polyphenols, which results in higher antioxidant activity.

Over the last few decades, the industrial community has had a massive push to utilize sustainable processing for the production of food [68]. According to the results, the aqueous UAE of the SBLs pretreated only with freeze-drying had the most optimal TPC and antioxidant activity. Thus, it could be proposed that extracting polyphenols from the freeze-dried sample using water, as a green solvent, and UAE, as a green extraction method, is an effective process that can increase the efficiency of the valorization of food by-products. UAE enhances the efficiency of the extraction process by having a shorter process time, better penetration, lower solvent consumption, and higher extraction yield. Therefore, UAE could be considered a sustainable alternative to CE methods [66,69]. In addition, water is a suitable extracting solvent broadly applied for the extraction of biologically active compounds [70]. Kumar et al. (2020) highlighted that when cold water was used to extract polyphenols from the lyophilized leaves of *Talinum triangulare* L., the obtained TPC was higher than that of other solvents [71].

## 3. Materials and Methods

### 3.1. Materials and Chemicals

The SBLs were of a sugar beet plant (*Beta Vulgaris* L., var. SMART DJERBA-KWS) aged 10 months (March–January 2021), cultivated at a local farm in Padova, Italy. The area and weight of the SBLs were about 20–30 cm^2^ and 2–3.5 g, respectively. They were transported to the laboratory in thermal boxes.

All the chemicals and solvents used in this work were of analytical grade. Sodium hydroxide, citric acid, sodium citrate tribasic dehydrate, sulfuric acid, hydrochloric acid, EtOH, MeOH, polyvinylpolypyrrolidone, triton X-100, 3,3′-dithiodipropionic acid, barium hydroxide, sodium acetate, potassium carbonate, sodium methoxide, acetonitrile, sodium carbonate, Folin–Ciocalteu reagent, gallic acid, Trolox, iron(III) chloride, 2,4,6-Tri(2-pyridyl)-s-triazine (TPTZ), and 2,2-diphenyil-1- picrylhydrazyl (DPPH) were bought from Sigma (St. Louis, MO, USA). AccQTag Ultra Derivatization Kit, and 6-aminoquinolyl-N-hydroxysuccinimidyl carbamate (AQC) were purchased from Waters (Milford, CT, USA). The water utilized in all the analyzes was deionized and distilled.

### 3.2. Sample Preparation

In preparing samples, all the steps were performed with high care to prevent any physical damage to the leaves. First, the SBLs were washed with distilled water to remove the soil, and their stalks were separated. Next, the cleaned SBLs were treated using four different methods, as shown in Figure 1. Mortar and pestle were used to grind the SBLs while frozen using LN before/after the freeze-drying process. Freeze-drying was performed under the pressure of 28 mbar at −50 °C for four days using a freeze dryer (Modulyo, Edwards, West Sussex, UK). All samples were vacuum-packed and stored at −18 °C until the following analysis.

### 3.3. Extraction of PPO from SBLs

The PPO was extracted in triplicate using the method described by Tinello et al. (2018) with some modifications [72]. Briefly, 1 g of leaves was added to 20 mL of 0.1 M sodium citrate buffer at pH 6.0 containing 0.10 g insoluble Polyvinylpolypyrrolidone (PVPP) and 500 µL Triton X-100. Then, the mixture was ultraturraxed (T-25 high-speed homogenizer, IKA-Werke GmbH and Co. KG, Staufen, Germany) at 1000× *g* in an ice bath for 2 min twice, with a 1-min interval in the middle. Then, the enzymatic extracts were centrifuged at 4000× *g* for 10 min at 4 °C and filtered through Whatman No. 1 filter papers and 0.45-μm syringe filter membranes. Finally, the extracts were refrigerated at −18 °C until the following analyses.

### 3.4. UAE and CE of Polyphenols from SBLs

**Figure 6:**
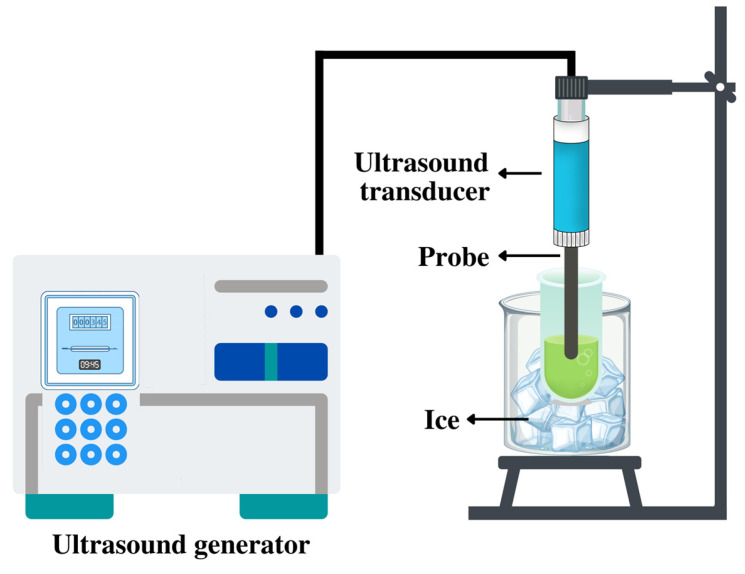
Schematic of UAE assembly with ultrasound probe system.

The extraction of polyphenols was performed according to the methods reported by Ebrahimi et al. (2022) with some minor modifications [57]. CE was carried out by macerating the leaves in EtOH:water 14:6 (*v*/*v*) for 24 h at 25 °C. UAE was performed using an ultrasound apparatus (HD 2200.2, Bandelin, Berlin, Germany) connected to a 6 mm titanium probe (KE 76, Bandelin, Berlin, Germany) which was immersed for 2 m in the mixture. Figure 6 shows the assembly of the used UAE system. The UAE conditions were as follows: 200 W output power, 25% amplitude, 20 kHz frequency, and 15 min extraction time. The extraction was performed in two consecutive cycles, and the combined extracts were used for further analysis. During the UAE, the extracting cell was placed in a beaker containing ice to prevent increasing the temperature. Distilled water/EtOH:water 14:6 (*v*/*v*) was used as the extracting solvent for UAE. For both UAE and CE methods, the solid:liquid ratio was 1:20 (*w*/*v*), and after the extraction processes, the obtained mixtures were centrifuged at 4000× *g* and 4 °C for 10 min. The extracts were filtered with Whatman No. 1 filters and stored at −18 °C until the analytical measurements. The extraction processes were performed in triplicate.

### 3.5. Analysis of Chemical Properties and PPO Activity of the Leaves

#### 3.5.1. Proximate Composition and Water Activity of the Leaves

Water activity was measured using a LabMaster.aw instrument (Novasina AG, Lachen, Switzerland) at 25 °C. The moisture, crude protein, crude fiber, and ash of SBLs were determined by the method described by Meng et al. (2022) [33]. The moisture content was determined using the oven-drying method at 105 °C for 24 h. Since the food matrices are concentrated after freeze-drying, the dry matter was calculated to report the results based on DM. Ash content was measured by weighing the samples before and after burning them in a muffle furnace at 550 °C for 6 h. The crude protein content was evaluated using the Kjeldahl method, and the results were multiplied by the nitrogen conversion factor of 6.25. The crude fiber was evaluated by boiling the leaves in 0.26 M sulfuric acid for 30 min. The obtained insoluble residue was filtered and washed, and the filtrate was boiled in 0.31 M sodium hydroxide and was filtered and rewashed. The final filtrate was dried at 130 °C for 120 min. The weight loss was measured at 350 °C. The crude lipid content was determined according to the Soxhlet technique using a Soxtec™ 2046 Extraction System (FOSS, Hillerød, Denmark).

#### 3.5.2. Determination of AA Profile

An Agilent 1260 Infinity HPLC (Agilent Technologies, Santa Clara, CA, USA), which was equipped with a reversed-phase column C18 (CORTECS C18, 2.7 µm, 2.1 × 150 mm) kept at 45 °C and a diode array detector (Agilent 1260 Series, DAD VL+), was used for the separation and quantification of amino acids in SBLs. AAs were analyzed after acid hydrolysis and pre-column derivatization with AQC, separated by RP-HPLC and analyzed by UV detection according to the method described by Bosch et al. (2006) [73]. An AccQTag Ultra Derivatization Kit was used for HPLC standards. Briefly, for the determination of alanine, arginine, aspartic acid, glutamic acid, glycine, histidine, isoleucine, leucine, lysine, methionine, phenylalanine, proline, serine, threonine, tyrosine, and valine, the protein of the sample was hydrolyzed with hydrochloride acid 6 M at 105 °C for 24 h. Cys was determined as the sum of cysteine and cystine after the reaction with 3,3′-dithiodipropionic acid, producing a mixed disulfide, which then underwent acid hydrolysis accordingly [74]. After hydrolysis, the samples were neutralized with sodium hydroxide 8 M, adjusted to volume, and filtered with 0.45 µm filters. The derivatization step was conducted by adding 70 µL of AccQ-Tag Ultra borate buffer and 10 µL of the filtered sample to a vial. Then, the solution was mixed by means of a vortex for several seconds. Next, 20 µL of the derivatization agent (dissolved in acetonitrile) was added, and the mixture was heated for 10 min at 55 °C. The sample was further diluted with 900 µL borate buffer and 5 µL was injected.

Tryptophan (Try) was determined following the method adapted from CD 2000/45/EC [75]. First, 100 mg of the sample was placed in a Teflon hydrolysis tube, then 8 g barium hydroxide and 16 mL water were added, and the tubes were incubated at 105 °C for 24 h. Hydrolysates were cooled down, neutralized to pH 7 using 6 N HCl, and diluted to 100 mL with 1 M sodium borate buffer. Aliquots of these solutions were filtered through a 0.22 µm syringe filter. A total of 20 µL of the sample was injected into the column (Xselect HSS T3, 5 µm; 4.6 × 250 mm), and the separation was performed by an isocratic elution system consisting of 25 mM sodium acetate/acetonitrile (91:9) delivered at 0.9 mL/min and detected with a DAD at 280 nm.

#### 3.5.3. Determination of FA Profile

The content of FAs in SBLs was analyzed using the two-dimensional gas chromatography technique (GC × GC), according to the research conducted by Ebrahimi et al. (2022) with some modifications [76]. For doing so, a gas chromatograph (GC) (Agilent 7890A, Agilent Technologies, Milan, Italy) equipped with an Agilent 7683 autosampler, a flame ionization detector (FID), and an Agilent CFT modulator was employed.

The sample preparation for FA analysis was carried out by adding 40 mg of the SBLs to a glass test tube containing 1 mL of sodium methoxide in MeOH (0.5 M). The resulting mixture was incubated at 50 °C for 15 min and cooled for 10 min at room temperature. After adding 1.5 mL MeOH containing 5% of HCl, the mixture was incubated at 80 °C for 15 min and cooled at room temperature for 10 min. Then, 2 mL hexane and 2 mL potassium carbonate 6% were added to the cooled mixture. The obtained solution was vortexed for 30 s and centrifuged at 4000× *g* at 4 °C for 5 min. The supernatant containing fatty acid methyl ester (FAME) was injected into the GC.

The GC temperature programming was as follows: initial oven temperature of 40 °C (2 min hold time), heating rate of 50 °C/min up to 170 °C (25 min hold time), temperature increase by 2 °C/min up to 250 °C (14 min hold time), injection port temperature of 270 °C, and a detector port temperature of 300 °C. The injected volume was 1 µL in a split mode (160:1). Hydrogen was used as the carrier gas. As the primary column, a Supelco SP-2560 (75 m × 0.18 mm × 0.14 µm film thickness) with a flow rate ranging from 0.25 to 0.28 mL/min at a rate of 0.0005 mL/min was used. An Agilent J&W HP-5 ms (3.8 m × 0.25 mm × 0.25 µm film thickness) with a flow rate ranging from 20 to 28 mL/min at a rate of 0.12 mL/min, was employed as the second column. The valves were set to a modulation delay of 1 min, a modulation period of 3.2 s, and a sampling time of 3.08 s. One microliter sample was injected in the pulsed split mode at a pressure of 25 psi for 18 s and a split ratio of 120:1. The split/splitless inlet was performed at 270 °C.

The resulting two-dimensional chromatograms were processed with comprehensive GC × GC software (GC Image R 2.2 GC × GC: Zoex Corp., Houston, TX, USA). The individual FAs were reported as the % of total fatty acids.

#### 3.5.4. Determination of Polyphenol Oxidase Activity

PPO activity was spectrophotometrically measured in the enzymatic extracts. The enzyme activity was detected according to the method described by Zocca et al. (2011) with some modifications [77]. The absorbance was recorded at 400 nm and 25 °C using a spectrophotometer (Varian Carry 50 Bio UV/Vis, Agilent Technologies, Santa Clara, CA, USA). The reaction sample included 1 mL of 10 mM catechol in sodium citrate buffer (0.1 M, pH = 6) and 10 μL of PPO extract. The blank sample contained 10 μL of PPO extract and 1 mL sodium citrate buffer (0.1 M, pH = 6) without the substrate. The absorbance was monitored continuously for 180 s considering the linear part of the PPO kinetic curve. For calculating the specific activity of PPO, one unit of enzyme activity was defined as the increase of 0.001 in absorbance per min, and the obtained value was divided by the protein content of PPO extracts, which was measured using the method described by Bradford (1976) [78].

### 3.6. Analysis of Phenolic Extracts

#### 3.6.1. Determination of TPC

The TPC was measured using Folin–Ciocalteu colorimetric method as described by Azuma et al. (1999) with some modifications [79]. In brief, 500 µL of the diluted extract was added to a mixture of 1.25 mL sodium carbonate solution 7.5% and 250 µL Folin–Ciocalteu reagent 50%. In the blank sample, 500 µL of the extracting solvent was used instead of the extract. After keeping the final mixtures for 30 min in a dark place at room temperature, the absorbance was recorded at 650 nm using the spectrophotometer. A standard curve of gallic acid (y = 10.132x–0.0124, R^2^ = 0.996) at concentrations ranging from 0 to 0.15 mg/mL was used to calculate and report results as mg GAE/g DM.

#### 3.6.2. Determination of Antioxidant Activity

The antioxidant activity of extracts was analyzed using the DPPH and FRAP methods. The DPPH free radical scavenging activity of extracts was evaluated by the method described by Massini et al. (2016) with some modifications [80]. Briefly, 200 µL of diluted extracts was added to 1.8 mL of a 0.1 mM DPPH˙ solution in EtOH 95%. For the blank sample, 200 µL of the extracting solvent was added instead of the extract. Then, the mixtures were kept at room temperature for 30 min, and the decrease in absorbance was read at 517 nm in the spectrophotometer. A standard curve of Trolox (y = 0.0093x − 0.0121, R^2^ = 0.999) at concentrations ranging from 0 to 0.1 mg/mL was employed for reporting the results as mg TE/g DM.

Ferric reducing antioxidant power (FRAP) was assayed based on the method described by Stratil et al. (2006) with some modifications [81]. The FRAP reagent was prepared by mixing 38 mM of anhydrous sodium acetate buffer (pH 3.6), 20 mM iron (III) chloride in Milli-Q water, and 10 mM of TPTZ in 40 mM HCl, in a ratio of 10:1:1. Then, 900 μL of prepared FRAP reagent was added to 100 μL of diluted extract/extracting solvent (blank). The mixture was vortexed for 1 min and incubated for 30 min at 37 °C. After incubation, the absorbance was recorded at 593 nm. A standard curve of Trolox (y = 0.0165x − 0.0022, R^2^ = 0.999) at concentrations ranging from 0 to 0.1 mg/mL was employed for reporting the results as mg TE/g DM.

### 3.7. Statistical Analysis

All the analyzes were carried out in triplicate (*n* = 3) for statistical analysis, and the results were expressed as mean ± standard. The data collected from the assays were processed using IBM SPSS Statistics (Version 20.0, SPSS Inc, Chicago, IL, USA).

Normality and homoscedasticity were tested using the Shapiro–Wilk and Levene’s tests, respectively. Where needed, the data were normalized by log_10_ transformation. A one-way Analysis of Variance (ANOVA) was used to analyze the results of proximate composition, water activity, AA and FA profiles, and PPO activity. Moreover, a two-way ANOVA was employed to process the data from TPC, DPPH, and FRAP tests. The significance level and confidence level were 0.05 and 95%, respectively. The comparisons were made using Tukey’s Honestly Significant Difference (HSD) test. Origin Pro 2022 (Northampton, MA, USA) was used for graphing the data.

## 4. Conclusions

Even though the proposed method could be considered a green extraction methodology, more research should be carried out on other food by-products to confirm its effectiveness on all plants. The most prominent results highlighted by the present study could be summarized as follows:

1. Combining aqueous UAE and freeze-drying techniques could be a potential tool for extracting polyphenols more efficiently, which may result from the inactivation of PPO by freeze-drying and the breakage of the cell membrane by UAE.
2. The freeze-drying method decreases the lipid content and increases the fiber content, essential fatty acids, and some essential amino acids and phenolic content of SBLs, making this by-product a potential dietary food supplement and a suitable substrate for fermentation.
3. The combined effect of aqueous UAE and freeze-drying techniques could be a circular economy approach enhancing the by-products’ valorization.

## Figures and Tables

**Table 1:** Proximate composition and water activity of SBLs with different treatments.

Component	Type of Leaves			
	FL ^1^	FD ^3^	NFD ^2^	FDN ^4^
Water activity	0.98 ± 0.00 ^c^	0.10 ± 0.00 ^b^	0.08 ± 0.00 ^a^	0.08 ± 0.00 ^a^
Moisture (%)	82.42 ± 0.08 ^b^	9.26 ± 0.33 ^a^	8.63 ± 0.18 ^a^	8.95 ± 0.60 ^a^
Dry matter (%)	17.58 ± 0.08 ^a^	90.74 ± 0.33 ^b^	91.37 ± 0.18 ^b^	91.05 ± 0.64 ^b^
Ash (g/100 g DM ^5^)	10.23 ± 0.13 ^a^	10.49 ± 0.18 ^a^	10.08 ± 0.08 ^a^	10.23 ± 0.25 ^a^
Crude protein (g/100 g DM)	31.31 ± 0.05 ^d^	27.76 ± 0.07 ^a^	28.29 ± 0.08 ^b^	28.75 ± 0.05 ^c^
Crude fiber (g/100 g DM)	2.13 ± 0.03 ^a^	4.04 ± 0.04 ^c^	3.76 ± 0.04 ^b^	3.77 ± 0.08 ^b^
Crude lipid (g/100 g DM)	1.16 ± 0.05 ^bc^	1.08 ± 0.08 ^b^	1.30 ± 0.09 ^c^	0.46 ± 0.10 ^a^

Data are reported as mean ± SD (*n* = 3). ^a, b, c, d^ Different letters show that there is a significant difference (*p* ≤ 0.05), according to one-way ANOVA and Tukey’s Honestly Significant Difference (HSD) tests; ^1^ Fresh SBLs; ^2^ Lyophilized SBLs ground without LN; ^3^ Lyophilized SBLs ground with LN before freeze-drying; ^4^ Lyophilized SBLs ground with LN after freeze-drying; ^5^ Dry matter.

**Table 2:** Amino acid profile of samples.

Amino Acids (% Total AAs)	Type of Leaves			
	FL	FD	NFD	FDN
Histidine	3.81 ± 0.16 ^a^	3.59 ± 0.00 ^a^	3.67 ± 0.01 ^a^	3.59 ± 0.08 ^a^
Arginine	5.45 ± 0.06 ^a^	5.54 ± 0.00 ^a^	5.43 ± 0.10 ^a^	5.44 ± 0.05 ^a^
Serine	5.63 ± 0.14 ^a^	5.62 ± 0.01 ^a^	5.80 ± 0.02 ^a^	5.71 ± 0.08 ^a^
Glycine	7.34 ± 0.37 ^a^	6.88 ± 0.01 ^ab^	6.71 ± 0.03 ^a^	6.73 ± 0.03 ^a^
Aspartic acid	9.89 ± 0.15 ^a^	10.24 ± 0.01 ^b^	10.86 ± 0.00 ^c^	10.79 ± 0.08 ^c^
Glutamic acid	14.32 ± 0.21 ^a^	14.49 ± 0.02 ^a^	15.92 ± 0.02 ^a^	14.50 ± 1.44 ^a^
Threonine	5.81 ± 0.54 ^b^	5.13 ± 0.01 ^ab^	4.98 ± 0.02 ^a^	5.23 ± 0.27 ^ab^
Alanine	5.57 ± 0.27 ^a^	5.82 ± 0.03 ^a^	5.73 ± 0.01 ^a^	5.86 ± 0.12 ^a^
Proline	4.85 ± 0.16 ^a^	4.77 ± 0.01 ^a^	4.66 ± 0.02 ^a^	4.70 ± 0.05 ^a^
Lysine	7.06 ± 0.46 ^a^	8.14 ± 0.01 ^b^	7.95 ± 0.01 ^b^	8.32 ± 0.38 ^b^
Methionine	1.29 ± 0.14 ^a^	1.38 ± 0.01 ^a^	1.28 ± 0.02 ^a^	1.23 ± 0.04 ^a^
Tyrosine	3.91 ± 0.19 ^b^	3.62 ± 0.01 ^a^	3.64 ± 0.01 ^a^	3.62 ± 0.01 ^a^
Valine	4.93 ± 0.19 ^ab^	5.12 ± 0.01 ^b^	4.64 ± 0.01 ^a^	4.91 ± 0.26 ^ab^
Cysteine + Cystine	1.54 ± 0.087 ^a^	1.44 ± 0.01 ^a^	1.39 ± 0.01 ^a^	1.46 ± 0.09 ^a^
Isoleucine	3.44 ± 0.29 ^ab^	3.78 ± 0.01 ^b^	3.26 ± 0.01 ^a^	3.54 ± 0.28 ^ab^
Leucine	7.88 ± 0.11 ^ab^	7.97 ± 0.00 ^b^	7.54 ± 0.01 ^a^	7.81 ± 0.27 ^ab^
Phenylalanine	5.09 ± 0.12 ^b^	4.91 ± 0.01 ^ab^	4.73 ± 0.01 ^a^	4.85 ± 0.13 ^a^
Tryptophan	2.19 ± 0.14 ^c^	1.54 ± 0.01 ^a^	1.82 ± 0.01 ^b^	1.70 ± 0.11 ^ab^

Data are reported as mean ± SD (*n* = 3). ^a, b, c^ Different letters show that there is a significant difference (*p* ≤ 0.05), according to one-way ANOVA and Tukey’s HSD test.

**Table 3:** Profile of FAs in the fresh and freeze-dried leaves.

	Fatty Acids (% of Total FA Content)	FL	FD	NFD	FDN
SFAs ^2^	Caproic acid (6:0)	0.37 ± 0.06 ^bc^	ND ^1^	0.50 ± 0.10 ^c^	0.24 ± 0.03 ^b^
	Enanthic acid (7:0)	0.18 ± 0.03 ^b^	ND	ND	0.01 ± 0.01 ^a^
	Caprylic acid (8:0)	0.09 ± 0.02 ^a^	0.34 ± 0.06 ^b^	2.72 ± 0.02 ^c^	0.32 ± 0.03 ^b^
	Pelargonic acid (9:0)	0.08 ± 0.02 ^b^	ND	ND	0.01 ± 0.01 ^a^
	Capric acid (10:0)	0.07 ± 0.04 ^a^	0.25 ± 0.04 ^b^	1.88 ± 0.04 ^c^	0.23 ± 0.02 ^b^
	Lauric acid (12:0)	0.11 ± 0.03 ^a^	0.29 ± 0.03 ^b^	1.41 ± 0.02 ^c^	0.38 ± 0.11 ^b^
	Myristic acid (14:0)	0.68 ± 0.03 ^b^	0.53 ± 0.05 ^a^	0.74 ± 0.06 ^b^	0.54 ± 0.04 ^a^
	Pentadecanoic acid (15:0)	0.34 ± 0.05 ^a^	0.24 ± 0.03 ^a^	0.27 ± 0.02 ^a^	0.27 ± 0.06 ^a^
	Palmitic acid (16:0)	27.87 ± 0.33 ^c^	15.97 ± 0.35 ^a^	15.83 ± 0.14 ^a^	18.40 ± 0.50 ^b^
	14-Methylhexadecanoic acid (17:0 *anteiso*)	0.03 ± 0.01 ^a^	0.03 ± 0.02 ^a^	ND	0.01 ± 0.01 ^a^
	Margaric acid (17:0)	1.60 ± 0.07 ^c^	1.45 ± 0.04 ^b^	1.41 ± 0.02 ^b^	1.17 ± 0.05 ^a^
	16-Methylheptadecanoic acid (18:0 *iso*)	0.02 ± 0.01 ^a^	0.04 ± 0.02 ^a^	0.02 ± 0.01 ^a^	0.03 ± 0.02 ^a^
	Stearic acid (18:0)	3.05 ± 0.09 ^c^	1.14 ± 0.09 ^b^	0.91 ± 0.04 ^a^	1.20 ± 0.02 ^b^
	Arachidic acid (20:0)	0.24 ± 0.03 ^a^	0.31 ± 0.16 ^a^	0.23 ± 0.03 ^a^	0.27 ± 0.09 ^a^
	Heneicosylic acid (21:0)	0.14 ± 0.02 ^b^	0.12 ± 0.03 ^b^	0.04 ± 0.01 ^a^	0.12 ± 0.03 ^b^
	Behenic acid (22:0)	0.69 ± 0.02 ^a^	0.61 ± 0.04 ^a^	0.57 ± 0.02 ^a^	0.66 ± 0.10 ^a^
	Tricosanoic acid (23:0)	0.34 ± 0.03 ^a^	0.23 ± 0.03 ^a^	0.23 ± 0.03 ^a^	0.30 ± 0.07 ^a^
	Lignoceric acid (24:0)	1.10 ± 0.10 ^a^	1.06 ± 0.02 ^a^	1.05 ± 0.01 ^a^	1.28 ± 0.27 ^a^
MUSFAs ^3^	Myristoleic acid (14:1 *cis*-9)	0.04 ± 0.01 ^b^	ND	ND	0.01 ± 0.01 ^a^
	*trans*-10-Pentadecenoic acid (15:1 *trans*-10)	0.00 ± 0.01 ^a^	ND	ND	0.02 ± 0.03 ^a^
	*trans*-6-Hexadecenoic acid (16:1 *trans*-6)	0.10 ± 0.03 ^b^	0.02 ± 0.00 ^a^	ND	0.05 ± 0.04 ^ab^
	*cis*-7-Hexadecenoic acid (16:1 *cis*-7)	1.58 ± 0.08 ^b^	0.86 ± 0.04 ^a^	0.91 ± 0.02 ^a^	0.68 ± 0.16 ^a^
	Palmitoleic acid (16:1 *cis*-9)	3.47 ± 0.22 ^d^	1.09 ± 0.04 ^b^	0.53 ± 0.03 ^a^	2.35 ± 0.20 ^c^
	*cis*-11-Hexadecenoic acid (16:1 *cis*-11)	0.01 ± 0.02 ^a^	ND	ND	ND
	*cis*-9-Heptadecenoic acid (17:1 *cis*-9)	1.29 ± 0.03 ^b^	0.85 ± 0.02 ^a^	0.94 ± 0.04 ^a^	0.85 ± 0.15 ^a^
	Elaidic acid (18:1 *trans*-9)	0.10 ± 0.02 ^b^	ND	ND	0.01 ± 0.02 ^a^
	Oleic acid (18:1 *cis*-9)	25.34 ± 0.39 ^c^	8.77 ± 0.23 ^a^	6.60 ± 0.28 ^a^	14.60 ± 2.96 ^b^
	Vaccenic acid (18:1 *cis*-11)	1.69 ± 0.05 ^c^	0.67 ± 0.06 ^b^	0.38 ± 0.08 ^a^	0.69 ± 0.04 ^b^
	*cis*-10-Nonadecenoic acid (19:1 *cis*-10)	0.17 ± 0.03 ^a^	0.12 ± 0.01 ^a^	0.14 ± 0.01 ^a^	0.14 ± 0.03 ^a^
	Gondoic acid (20:1 *cis*-11)	0.28 ± 0.03 ^b^	0.19 ± 0.01 ^a^	0.20 ± 0.02 ^a^	0.28 ± 0.02 ^b^
	Gerucic acid (22:1 *cis*-13)	0.27 ± 0.02 ^b^	0.03 ± 0.02 ^a^	ND	0.06 ± 0.04 ^a^
	Nervonic acid (24:1 *cis*-15)	0.00 ± 0.01 ^a^	ND	ND	0.02 ± 0.03 ^a^
PUSFAs ^4^	*cis, cis*-6,9-Hexadecadienoic acid (16:2 *cis, cis*-6,9)	0.02 ± 0.01 ^a^	0.10 ± 0.02 ^bc^	0.12 ± 0.02 ^c^	0.06 ± 0.03 ^ab^
	All *cis*-7,10,13-Hexadecatrienoic acid (16:3 all *cis*-7,10,13)	0.32 ± 0.04 ^a^	1.31 ± 0.02 ^b^	1.23 ± 0.05 ^b^	0.79 ± 0.55 ^ab^
	trans, *cis*-8,13-Octadecadienoic acid (18:2 *trans, cis*-8,13)	0.07 ± 0.03 ^b^	0.02 ± 0.02 ^a^	ND	0.02 ± 0.01 ^a^
	Linoleic acid (18:2 *cis, cis*-9,12)	15.15 ± 0.42 ^a^	19.30 ± 0.06 ^b^	22.86 ± 0.04 ^c^	19.82 ± 1.43 ^b^
	γ-Linolenic (18:3 all *cis*-6,9,12)	0.10 ± 0.02 ^a^	0.19 ± 0.04 ^b^	0.13 ± 0.02 ^ab^	0.11 ± 0.04 ^b^
	Dihomo-γ-Linolenic acid (DGLA) (18:3 all *cis*-8,11,14)	0.01 ± 0.01 ^a^	0.06 ± 0.02 ^b^	0.04 ± 0.01 ^ab^	0.02 ± 0.02 ^a^
	α-Linolenic acid (18:3 all *cis*-9,12,15)	12.70 ± 0.20 ^a^	43.65 ± 0.15 ^d^	37.97 ± 0.04 ^c^	33.70 ± 1.90 ^b^
	Stearidonic acid (18:4 all *cis*-6,9,12,15)	0.03 ± 0.01 ^a^	0.02 ± 0.01 ^a^	0.02 ± 0.01 ^a^	0.02 ± 0.01 ^a^
	*cis, cis*-11,14-Eicosadienoic acid (20:2 *cis, cis*-11,14)	0.03 ± 0.01 ^a^	0.03 ± 0.01 ^a^	0.04 ± 0.00 ^a^	0.10 ± 0.08 ^a^
	All *cis*-8,11,14-Eicosatrienoic acid (20:3 all *cis*-8,11,14)	0.03 ± 0.01 ^b^	ND	ND	ND
	All *cis*-11,14,17-eicosatrienoic acid (20:3 all *cis*-11,14,17)	0.02 ± 0.01 ^a^	0.04 ± 0.02 ^ab^	0.04 ± 0.01 ^ab^	0.05 ± 0.00 ^b^
	Arachidonic acid (20:4 all *cis* 5,8,11,14)	0.13 ± 0.02 ^b^	0.04 ± 0.00 ^a^	0.03 ± 0.01 ^a^	0.06 ± 0.02 ^a^
	Cetoleic acid (22:2 *cis*-11)	0.01 ± 0.01 ^a^	0.01 ± 0.00 ^a^	0.02 ± 0.00 ^a^	0.03 ± 0.02 ^a^
	Osbond acid (22:5 all *cis*-4,7,10,13,16)	ND	0.01 ± 0.00 ^b^	ND	0.02 ± 0.00 ^c^
	Cervonic acid (22:6 all *cis*-4,7,10,13,16,19)	0.02 ± 0.01 ^b^	ND	ND	ND

Data are reported as mean ± SD (*n* = 3). ^a, b, c, d^ Different letters show that there is a significant difference (*p* ≤ 0.05), according to one-way ANOVA and Tukey’s HSD test; ^1^ Not detected; ^2^ Saturated fatty acids; ^3^ Monounsaturated fatty acids; ^4^ Polyunsaturated fatty acids.

## Data Availability

Data are contained within the article.
